# Crossed hands strengthen and diversify proprioceptive drift in the self-touch illusion

**DOI:** 10.3389/fnhum.2014.00422

**Published:** 2014-06-17

**Authors:** Kenri Kodaka, Yuki Ishihara

**Affiliations:** Graduate School of Design and Architecture, Nagoya City UniversityNagoya, Japan

**Keywords:** self-touch illusion, proprioceptive drift, multi-sensory integration, body image, cognitive dissonance

## Abstract

In the self-touch illusion (STI), some can feel that both hands are touching each other even when they are separated actually. This is achieved by giving synchronized touches to both hands. Because the STI involves both hands (an administrating hand and a receptive hand) of a single person, two types of proprioceptive drifts (PDs) simultaneously occur in such a way that both hands are attracted to each other. It is known that the PD distance is generally larger for the administrating hand than for the receptive hand when the two hands are uncrossed. However, it remains unclear why such an asymmetrical relationship is observed universally. In this study, we conducted two types of experiment to induce the STI. The first experiment involved four conditions combining a factor of “whether the hands are uncrossed or crossed” and a factor of “whether the administrating hand is resting or active on the surface,” with the receptive (left) hand located at the body's midline. The result demonstrated that crossing hands and resting on surface (ROS) induced the STI. Specifically, crossing hands enhanced the amount of PD distance by more than two or three times. Moreover, it is interesting that strong PD with dominance of the receptive hand, which did not appear in the uncrossed condition, was observed frequently. The second experiment collected seven “illusion-sensitive” participants from the first experiment, all of whom had a strong tendency to feel the self-touch, and examined the effect of the location of the body midline on the PD when hands are crossed with the administrating hand ROS. The result demonstrated that the dominant hand on the PD completely differed among participants, but was relatively stable over the midline position and time in the same person. We also found that a small number of participants exhibited quite a different pattern of the PD in the identical posture. On the basis of the results, we analyze in detail how the dominant hand on the PD is determined in the STI.

## 1. Introduction

The self-touch illusion (STI), involving a proprioceptive sense and a sense of touch, was originally reported by Ehrsson et al. (2005). They originally named the STI the “somatic rubber hand illusion” because it has been sometimes regarded as a variation of the rubber hand illusion (RHI). Although some people exhibit a striking STI, far fewer studies have explored the STI than the RHI. In the RHI (reported first by Botvinick and Cohen, 1998), one feels as if one has a prosthetic hand as a part of one's body when an experimenter simultaneously strokes one's occluded hand and the prosthetic hand in one's sight (placed near the occluded hand in the same direction), where each stroked location is identical anatomically. The RHI integrates a proprioceptive sense, a sense of touch and a vision into a single physical event in such a way that the vision information is strongly weighted among the three types of senses. The best proof for this is that the proprioceptive sense for the occluded hand moves toward the prosthetic hand in sight by a specific distance during the illusion. This phenomenon has been called proprioceptive drift (PD). Several studies have demonstrated that the PD positively correlates with an agreement rating score on a questionnaire ownership statement like “I felt as if the rubber hand was my own hand” (e.g., Longo et al., 2008; Lopez et al., 2010; Kalckert and Ehrsson, 2012). It is instructive to confirm that the other hand (lacking the experimenter's stroke) plays no role whatsoever in inducing the RHI.

The participant's vision is thus essentially involved in the RHI, whereas the STI is induced with the participant's eyes blinded. A process of one hand's being stroked by an experimenter in the RHI also applies to the STI. In contrast, the stroke to the prosthetic hand in the RHI, which is non-sensical for a blinded person, is replaced with a participant stroking the prosthetic hand with the other hand. We call the stroked hand the “receptive hand” and the stroking hand the “administrating hand,” following the terminology of White et al. (2011). The stroke with the administrating hand should be simultaneous with the stroke on the receptive hand, and the two strokes should be in the same place anatomically. Therefore, the blinded participant's administrating hand's movement has been generally guided by the experimenter in inducing the STI (Ehrsson et al., 2005; White et al., 2011; Petkova et al., 2012; Aimola Davies et al., 2013). The STI thus induced is described as integration between the proprioceptive sense and the sense of touch. The simultaneity between the two types of stroking for both hands provides positive evidence for the self-touch situation. In contrast, the difference of the touch sense caused by texture and pressure and the difference between two hand positions in the proprioceptive sense negatively affect inducing the STI. The former can be reduced by covering both hands with identical material such as rubber gloves or by training the experimenter's skills. In contrast, the difference between the felt locations of both hands strongly obstructs the STI. Conversely, success in inducing the STI strongly depends on how the difference between two proprioceptive senses is deceived successfully. Actually, strong STI reduces the sense of location difference, leading a participant to feel that both hands are in a closer position. This phenomenon is a PD in the STI.

Unlike the RHI, the PD in the STI occurs in such a way that two types of sense of hand locations attract each other. Accordingly, the PD of both the receptive and the administrating hands should be considered in the STI. White et al. (2011) first described a power balance between two PDs attracting each other in the STI, demonstrating that the PD distance is generally larger for the administrating (right) hand than for the receptive (left) hand. In this experiment, the stroke stimulation was conducted for each receptive hand and prosthetic hand via a writing brush. Each participant was instructed to hold the handle of the brush in the administrating hand, as the experimenter guided the brush movement by manipulating the upper part of the handle. As their study reported, it is probable that the proprioceptive sense of the administrating hand relatively decreased in reliability because, first, holding something in the air is unstable for the hand and, second, the experimenter substantially controlled the participant's administrating hand. Lack of proprioceptive sense reliability for a body part more greatly alters body part's location sense and induces acceptance of a potentially reasonable body image in the neighbor's space. The foregoing deduction is simply summarized as follows: a decrease in the reliability of one hand's proprioceptive sense may increase that hand's PD. We call that principle the “proprioceptive reliability paradigm” hereafter [This is consistent with the Maximum Likelihood Estimation (MLE) model, Ernst and Bülthoff (2004) for a review]. The proprioceptive reliability paradigm is consistent with the admin(istrating)-dominant drift found in their study. Admin-dominant asymmetry of the PD was reproduced in Aimola Davies et al. (2013) with the identical experimental set when the distance between two hands (uncrossed) ranges from 15 to 60 cm.

The hypothesis based on the proprioceptive reliability paradigm that a difference of the reliability of the proprioceptive sense between two hands causes asymmetrical PD seems strongly appealing and reasonable although little evidence exists to demonstrate the validity of the hypothesis because of the limited number of related studies. Therefore, it is highly important to examine how certain factors that potentially involve a change of proprioceptive sense reliability influence the PD's power balance. Recently, Rincon-Gonzalez et al. (2011) showed that a proprioceptive reliability of the arm is mapped uniquely to each individual but has a general trend to decrease if the corresponding hand locates at a contralateral location in their experiment where each eye-closed participant was first guided to reach their index finger to point at a specified area on a horizontal table and return to the resting position, and then to verbally estimate the 2D position of the finger pointed with their eyes open. Crossing hands inevitably makes at least one hand across a body midline, where shifting the body midline placement can control which hand(s) is (are) located in the contralateral region. The proprioceptive reliability paradigm supports the hypothesis that the PD of the contralateral hand is strengthened in such a situation. Thus, whether hands are crossed is a good factor to examine the validity of this paradigm. Their experiment also showed that a tactile feedback during the reaching operation improved the estimation performance (The similar effect has been reported in several papers, e.g., Rao and Gordon, 2001; Rabin and Gordon, 2004). In general STI studies, the administrating hand may relatively lack the tactile feedback because the administrating finger can contact the surface (of rubber hand), but the palm of the hand is not resting on any surface during the contact operation while the receptive hand completely remains resting on the surface. It is worth assuming that the admin-dominant drift is simply due to the asymmetrical situation where only the receptive hand gets sufficient tactile feedback. Thus, examining the effect of the administrating hand's resting on surface on the PD's power balance is also meaningful.

The present study conducted two experiments, wherein the right hand is the administrator of touch and the left hand the receptor. Based on the above discussion, the experiments analyzed the effects of three factors on an agreement rating for an illusion statement and the magnitude and the power balance of the PD: the first factor is whether hands are uncrossed or crossed, the second is whether the administrating hand is resting or active on the surface, and the third is the body midline placement. Interestingly, certain elements of the experimental results completely deny the conventionally reported admin-dominant asymmetry of the PD. The discussion will explore whether the proprioceptive reliability paradigm is valid for interpreting a mechanism that generates the asymmetrical PD by analyzing the results in detail.

## 2. Material and methods

### 2.1. Participants

Thirty-six right-handed students participated in Experiment I (22 females and 14 males, 19–33 years, *M* = 21.6). Seven “illusion-sensitive” students selected from 36 participants in Experiment I were requested to participate in Experiment II, whose score on the illusion strength in Experiment I surpassed a certain threshold (explained in section 2.7), all of whom complied with the request (2 females and 5 males, 19–23 years, *M* = 21.0). All participants were recruited from the Faculty of Design and Architecture, Nagoya-city University, and signed the informed consent prior to participation in each experiment. All participants received a book of tokens as compensation (Experiment I: 1000 Yen, Experiment II: 2000 Yen). The protocol was approved by the ethics committee of Nagoya-city University.

### 2.2. Apparatus

Each participant was seated on a chair behind a desk, and an experimenter sat on a chair on the other side of the desk to direct the experimental procedure. A rubber finger made of a cotton-filled fingertip (5 cm in length and 2 cm in diameter) was attached with tape along the midline of the table. A disk-shaped metallic vibrator (13,000 rpm, 12 mm in diameter, and 3.4 mm in thickness, T.P.C.) placed under a force-sensitive resistor (FSR, InterLink Electronic) made of a thin polymer film (7.6 mm in diameter) was attached inside the upper tip of the rubber finger. Thus, the rubber finger is entirely elastic, excluding the tip area in which the disk-shaped metal is embedded, representing the feeling of a nail's hardness. The vibrator and the FSR were connected to the Arduino Uno (open-source ATmega328 based microcontroller board) with cables to generate the vibration at the moment the FSR receives pressure. The mechanism allows one to feel the vibration on the contact surface when one presses the tip of the rubber finger. The distance from the near side of the desk to the contact point of the rubber finger was 10 cm. Another disk-shaped metallic vibrator connected with a pin of the Arduino Uno through cables was attached by tape on the nail of each participant's left index finger (covered with tape beforehand). The vibrator on the left index finger was also programmed to vibrate when the FSR was pressed. Via this mechanism, pressing the tip of the rubber finger by the right index finger simultaneously generates uniform vibrations to both the ball of the right index finger (of the administrating hand) and the nail of the left index finger (of the receptive hand).

### 2.3. Procedure

#### 2.3.1. Experiment I

**Figure 1:**
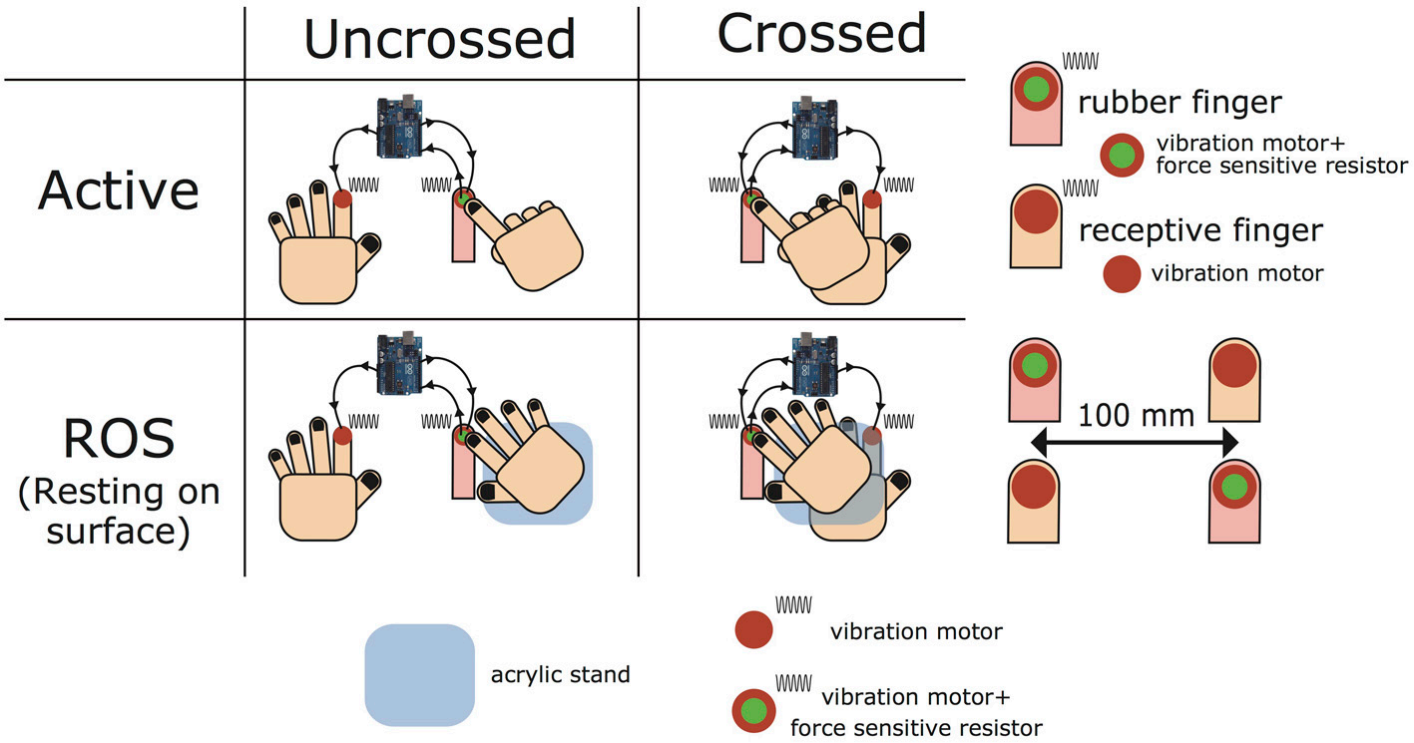
**Four types of hand postures in Experiment I**. There were four types of hand postures in Experiment I. Uncrossed/crossed determines whether hands are crossed or uncrossed with a distance of 10 cm. In active condition, the rubber finger was pressed by a right index finger while the rest of the four fingers in the right hand was wrapped, and the right elbow was kept in the air so that the right hand would not touch the upper surface of the table (Uncrossed × Active) or the back of the left hand (Crossed × Active). In ROS condition, an acrylic stand (30 mm in height) was placed on the right side of the rubber finger so that the palm of the right hand can rest on surface. The stand can separate the palm of the right hand from the back of the left hand at a short distance in Crossed × ROS.

**Figure 2:**
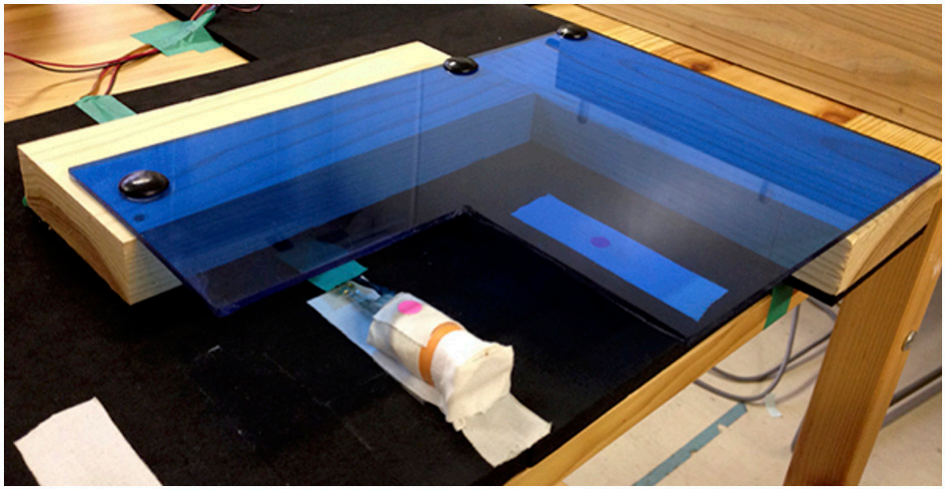
**Picture of rubber finger and acrylic stand set in the ROS condition**. An acrylic stand (30 mm high) was placed on the right side of the rubber finger so that the palm of the right hand could rest on the surface. In Experiment I, when the Active/ROS factor changed, the acrylic stand was located on the desk (ROS) or removed from the desk (Active). In Experiment II, the stand remained located on the desk during all sessions.

Experiment I induced the STI with four types of hand postures combining the following two factors (Figure 1). The first factor was determining whether hands are uncrossed (“Uncrossed condition”) or crossed (“Crossed condition”). In both conditions, the right index finger was placed on the tip of the rubber finger while the left index finger was placed 10 cm leftward (Uncrossed) or rightward (Crossed) from the tip of the rubber finger with the palm of the left hand resting on surface (ROS). In addition, the chair location was adjusted so as to place the left index finger along the body midline. The second factor was determining whether the palm of the right hand is ROS (“ROS condition”) or active on the surface (“Active condition”). In the active condition, each participant was directed to wrap the right four fingers excluding the index finger (touching the rubber finger) and keep the right elbow in the air so that the right hand would not touch the table's upper surface (Uncrossed) or the back of the left hand (Crossed). In ROS condition, an acrylic stand (30 mm high) was placed on the right side of the rubber finger so that the palm of the right hand could rest on the surface (Figure 2). The stand separated the palm of the right hand from the back of the left hand at a short distance in Crossed × ROS.

**Figure 3:**
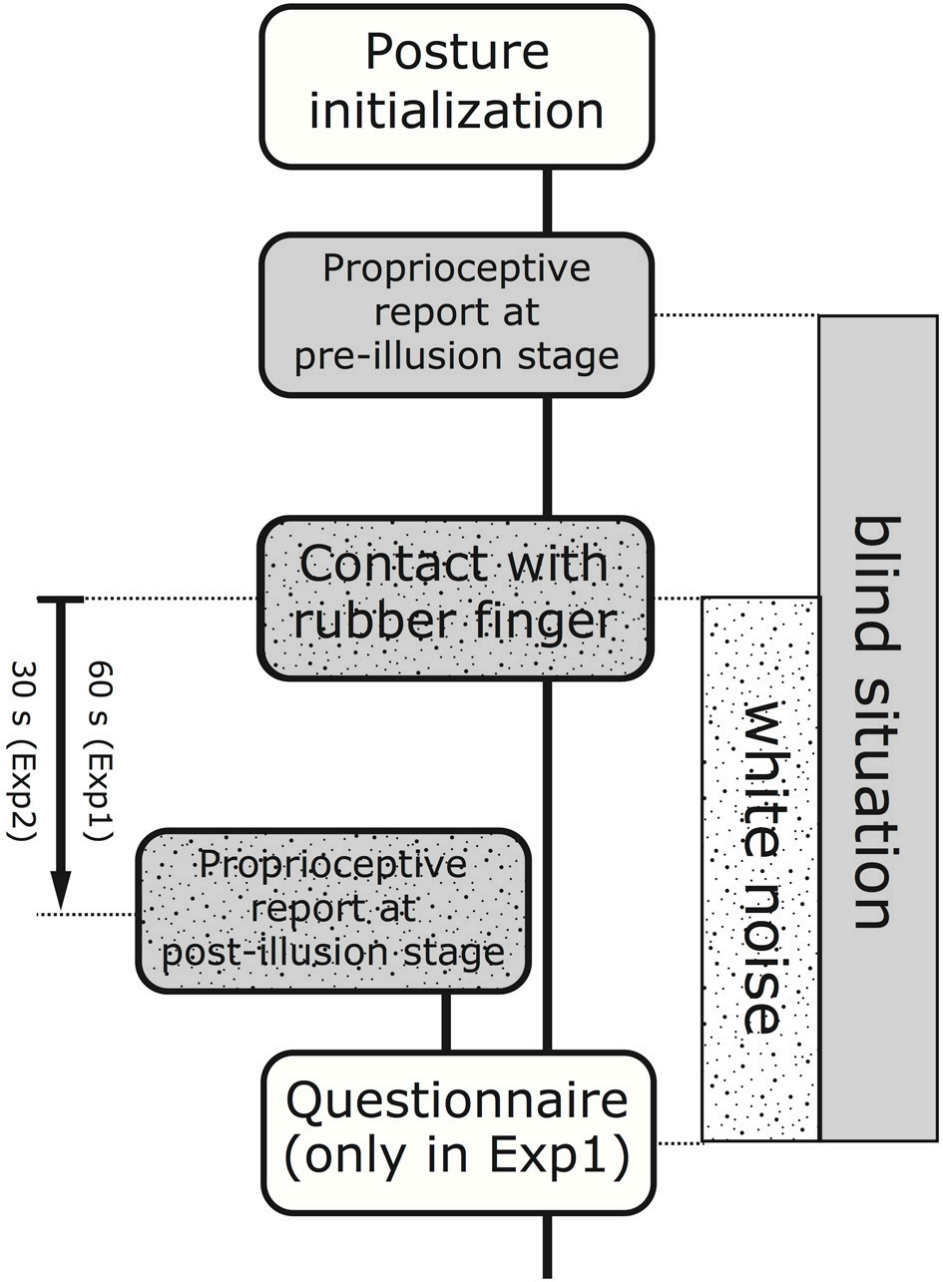
**Experimental procedure in one session**. One session comprises “Posture initialization,” “Proprioceptive report at pre-illusion stage,” “Contact with rubber finger,” “Proprioceptive report at post-illusion stage,” and “Questionnaire” in order, where “Proprioceptive report at post-illusion stage” begins without interrupting “Contact with rubber finger.” Each participant engaged in the “Proprioceptive report at pre-illusion stage” with eyes closed and “Contact with rubber finger” (including “Proprioceptive report at post-illusion stage”) with eyes closed and while hearing white noise through a wireless headphone. “Questionnaire” was replaced with a simple operation of counting to 5 s in one's head with both hands folded on one's lap in Experiment II. The length of time from the beginning of the “Contact with rubber finger” until the beginning of “Proprioceptive report at post-illusion stage” was 60 s in Experiment I but 30 s in Experiment II.

Experiment I comprised eight sessions, each involving one of four conditions and taking roughly 2 min. A series of eight conditions were determined at random with the following three constraints: (1) the first or second half (of four sessions) must include all four conditions; (2) the same condition should not be applied twice in a row; (3) all four conditions should be equally allocated during the first session (36/4 = 9). In this way, each of the four posture conditions includes two sessions (“1st session” and “2nd session”) for every participant. As Figure 3 depicts, each session started with “Posture initialization” where the experimenter instructed a participant to initialize his/her posture according to a selected condition. When the Crossed/Uncrossed factor changed, the chair was shifted rightward (Crossed) or leftward (Uncrossed) so that the left index finger was located along the body midline. When the Active/ROS factor changed, the acrylic stand was located on the desk (ROS) or removed from the desk (Active). Then, the participant rested the palm of the left hand on the desk, where the left index finger was placed on the left side (Uncrossed) or the right side (Crossed) of the rubber finger by 10 cm. Next, the participant was instructed to keep the right index finger touching (but not pressing) on the tip of the rubber finger while keeping the remaining four fingers wrapped (Active) or the palm of the right hand was resting on the stand (ROS). After the participant was instructed to close his/her eyes, they horizontally moved their head (leftward or rightward) with the head facing forward to align the center of the face along a horizontal plane where they feel the left or right index finger is located. An experimenter's touch on the back of the participant's left or right hand was a signal to begin the head movement toward the left or right index finger (“Proprioceptive report at pre-illusion stage”). In this operation, a small camera installed on the opposite side of the desk recorded the horizontal position (x) of the participant's nose in the camera coordinate system, where the participant was instructed to say Yes (“Hai” in Japanese) if the participant fixed his/her head position. After this operation was used for the left and right finger in sequence, the participant was instructed to realign the head at the body midline. Subsequently, the eye-closed participant started pressing the tip of the rubber finger with white noise playing through a wireless headphone as a signal (“Contact with rubber finger”). At 60 s after the beginning of “Contact with rubber finger,” the participant started again to report the felt location of each index finger while hearing the white noise, where the experimenter's touch on the back of each hand was again the signal for that operation (“Proprioceptive report at post-illusion stage”). It is notable that the operation described should be employed without suspending the work of pressing the rubber finger. The experimenter paused the white noise after recording two types of the camera positions, and subsequently the experimenter instructed the participant to realign the head at the body midline and fold his/her hands on their lap; finally, the participant was allowed to open their eyes. At the final stage in the session, the participant orally provided an agreement rating for five types of statements in a questionnaire (“Questionnaire”).

#### 2.3.2. Experiment II

Experiment II fixed each of the two factors at Crossed × ROS, while adding a new factor concerning the body midline alignment as a variable, including three conditions: the body midline is aligned with the administrating (right) hand (“Admin-centered condition”) at the middle of both hands (“Symmetric condition”), or with the receptive (left) hand (“Receptor-centered condition”). Note that Experiment I always adopted the Receptor-centered condition for this factor.

The participants repeatedly performed 15 sessions where each of the three condition was continued five times in a row; that is, the midline alignment was renewed at the beginning of the 6th and 11th sessions. The order of the body midline alignment was allocated at random for each participant, but two participants out of seven inevitably had the same order. The Experiment II procedure was largely identical with that of Experiment I, but without the questionnaire, and the time from the beginning of the “Contact with rubber finger” until the beginning of “Proprioceptive report at post-illusion stage” was shortened to 30 s (Figure 3). As in Experiment I, participants were allowed to open their eyes after the proprioception report at the post-illusion stage. If the session was not final (15th), participants were instructed to count 5 s in their heads with their hands on their lap instead of answering the questionnaire, and then, the posture was initialized with hands crossed and the palm of the right hand resting on the acrylic stand (Crossed × ROS), which was at the beginning stage of each session.

### 2.4. Initial instruction

Prior to the beginning of each experiment, the experimenter attached the vibration motor to the nail of the left index finger with tape after the participant was equipped with the wireless headphone and a mask whose center was marked by a black dot (for recording the nose position). In Experiment I, the experimenter explained the process of the experiment to each participant, taking roughly 10 min altogether. At that time, all the experimental equipment on the table was visible to the participant. During the instruction, the participant practiced to move his/her head horizontally few times where the experimenter asked to keep his/her head still for at least more than 2 s before saying Yes to prevent the measurement fluctuation in the “Proprioceptive report at pre/post-illusion stage.” In addition, the participant had an opportunity to press or release the tip of the rubber finger as a trial with their eyes open, enabling the participant to clearly understand that the pressing action caused simultaneous vibrations on the rubber finger and the left index finger. The experimenter told each participant that they might feel as if the right index finger touched the left index finger by pressing the rubber finger, and requested that they concentrate on exploring a unique way to press (and release) the rubber finger to induce that illusion as strongly as possible. Thus, it was the participant's responsibility to determine how long and strongly the rubber finger was pressed at each trial. To prevent a bias toward sensing the illusion, the experimenter also stated definitely at that time that some people cannot perceive such a feeling whatsoever. Finally, the participant tried to press the tip of the rubber finger with their eyes closed for 1 min in Uncrossed × Active as a final rehearsal. In Experiment II, a 1 min rehearsal of touching the rubber finger was conducted in the Receptor-centered condition (Crossed × ROS) after a general review of Experiment I.

### 2.5. Measurement of proprioceptive drift

In the studies of White et al. (2011) and Aimola Davies et al. (2013), the proprioceptive hand positions were recorded by orally reading the number on a ruler placed on top of a visual divider concealing the participant's hands below. To the best of the authors' knowledge, this is the only instance of measurement of two proprioceptive hand positions reported in STI studies. Such a measurement involves the sense of sight in the participant although the visual sense is absent in the STI. This approach indicates that the cognitive condition during the period of measurement by reading the number off the ruler would differ from that in the middle of the illusion. The present experiment employed an original measurement for recording two proprioceptive hand positions where the participant horizontally moves their head to the position aligned with the targeted finger. This approach enables the unseeing participant in the middle of the illusion to report the proprioceptive sense while continuing to press the rubber finger, without disrupting the illusion state.

To reduce the effect of camera lens distortion, the camera was placed along the rubber finger's extension in Experiment I (using both Crossed and Uncrossed conditions), but 5 cm leftward from the rubber finger in Experiment II (using only the Crossed condition). As previously stated, the participant's nose position was recorded by the camera tracking the black dot marker on the mask the participant wore. The preliminary measurement found that 5 cm horizontal width in the real world corresponded to 20 pixels around the center of the camera coordinate system (CCS) and to 19 pixels roughly 10 cm rightward or leftward from the center of the CCS. Thus, the measurement can distinguish the distance difference as small as 2.5–2.6 mm in the region that includes two fingers. Because the lens distortion effect in the measurement range was at most 5% in Experiment I and less than 2% in Experiment II, the authors ignored the correction operation in the following calculation.

The experimenter recorded the horizontal position (x) of the participant's head, which was displayed on a laptop computer in real time after they were averaged for the last 1 s (30 frames). If the positions were not stabilized when the participant said “Yes,” the experimenter urged to stabilize the head position by touching on a back of the corresponding hand again. The set of x coordinates for the receptive finger and the administrating finger is defined as (*A*_*x*_, *R*_*x*_) at the pre-illusion stage and (A^*^_x_, R^*^_x_) at the post-illusion stage. As a pre-processing step using the aforementioned measurements, the distance between the two hands at the pre-illusion stage (*D*_*pre*_) and the amount of the PD for the administering hand and the receptive hand (*A*_*drift*_, *R*_*drift*_, respectively) are calculated as follows, noting that the x axis in the CCS advances from right to left relative to the participant's body.

(1) $$D_{pre}=\{\begin{array}{ll} R_{x}-A_{x} & \;(Uncrossed)\\ A_{x}-R_{x} & \;(Crossed) \end{array}$$

(2) $$A_{drift}=\{\begin{array}{ll} A_{x}^{*}-A_{x} & \;(Uncrossed)\\ -(A_{x}^{*}-A_{x}) & \;(Crossed) \end{array}$$

(3) $$R_{drift}=\{\begin{array}{ll} -(R_{x}^{*}-R_{x}) & \;(Uncrossed)\\ R_{x}^{*}-R_{x} & \;(Crossed) \end{array}$$

#### 2.5.1. Attractivity and directivity

**Figure 4:**
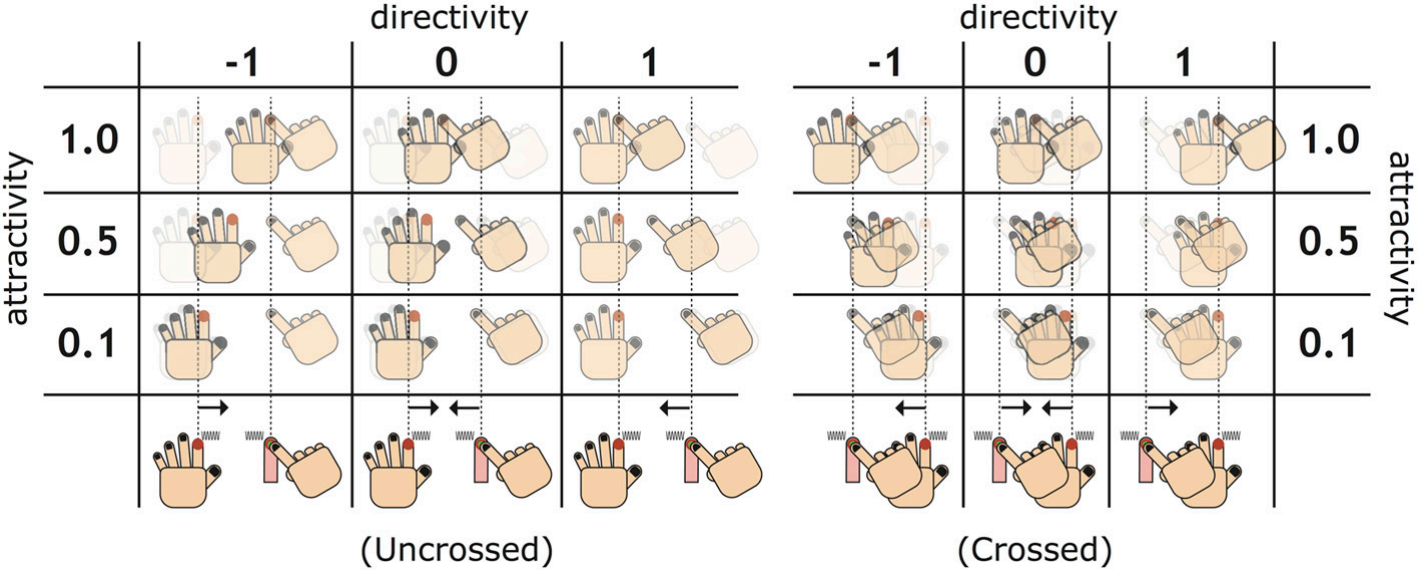
**Diagram for understanding new measures: attractivity and directivity**. The attractivity indicates how strongly two fingers are attracted after experiencing an illusion stage. The directivity measures the power balance of the attraction, focusing on the difference between the two types of proprioceptive drifts (PDs), whose sign is positive/negative if the PD of the administrating hand is larger/smaller than that of the receptive hand.

On the basis of the three variables defined above, the following calculation designed new measures called “attractivity” and “directivity,” representing the PD (Figure 4).

(4) $$attractivity\;=\frac{A_{drift}+R_{drift}}{D_{pre}}$$

(5) $$directivity\;=\frac{A_{drift}-R_{drift}}{D_{pre}}$$

The attractivity indicates how strongly two fingers are attracted by exposure to the illusion. The attractivity is zero when the distance between the two fingers does not change at the post-illusion stage, and one when the administrating finger completely sticks to the receptive finger. Furthermore, the attractivity is greater than one when crossed hands become uncrossed or uncrossed hands become crossed, and less than zero when the distance between the two fingers expands.

Directivity is defined as a measure of the power balance between the two types of the PDs. As the numerator in the right side of Equation (5) is obtained from subtracting the receptor's PD from the administrator's PD, directivity becomes positive when the administrator's PD dominates the receptor's PD, and negative when the receptor's PD dominates the administrator's. In addition, the directivity's intensity (absolute value) increases or decreases when the difference of the distance between two types of PDs increases or decreases, respectively. It is notable that large attractivity does not necessarily result in large directivity. As possibly strange, the negative receptor's PD increases the directivity whereas the negative administrator's PD decreases the directivity. This is consistent with an interpretation that one hand is pushed out from the other hand's (positive) drift. Thus, the negative PD of one hand is regarded as an indirect effect of the positive PD of the other hand in determining directivity.

### 2.6. Agreement ratings

In Experiment I, the participants were asked to complete a verbally administered questionnaire in which they rated their experiences of the following five perceptual effects conventionally adopted in previous STI studies (Ehrsson et al., 2005; White et al., 2011; Aimola Davies et al., 2013).

1. I felt as if I was touching my left index finger with my right index finger.
2. I felt as if I had more than one left index fingers or left hands.
3. I felt as if my left hand or left index finger was larger than normal.
4. I felt as if my left hand or left index finger moved.
5. I could no longer feel my left hand or left finger.

Statement 1 was the illusion statement and statements 2–5 originally served as controls for suggestibility and task compliance. The strength of each perceptual effect was rated using a seven-point scale (0 = not at all; 1 = slightly agree; 3 = moderately agree; 5 = strongly agree; 6 = very strongly agree) as in the study of Aimola Davies et al. (2013). The order of the five statements asked was randomized beforehand.

### 2.7. Participant recruitment in experiment II

The candidates for the “illusion-sensitive” participants in Experiment II were selected on the basis of the following two requirements.

1. An average of the attractivity in the Crossed condition (including 4 sessions) was more than 0.7.
2. An average of the agreement rating for Statement 1 in the Crossed condition was more than 4.0.

## 3. Results—experiment I

### 3.1. Attractivity

**Figure 5:**
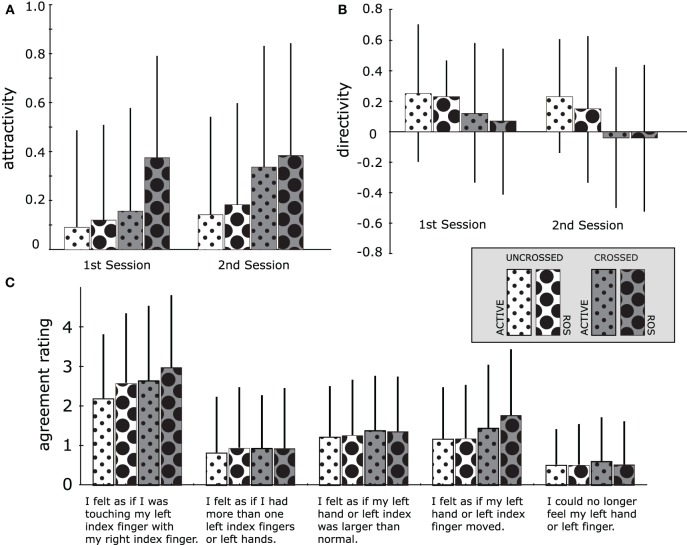
**Results of attractivity, directivity, and agreement rating for four types of hand postures (Uncrossed × Active, Uncrossed × ROS, Crossed × Active, Crossed × ROS) in Experiment I**. Charts **(A,B)** depict an average of attractivity/directivity among 36 participants at every first or second session. Chart **(C)** depicts an average of the questionnaire's agreement rating for five statements among 36 participants, each of which participated in two sessions for every posture. Error bars in all charts indicate standard error.

Figure 5A plots an average of the attractivity at every 1st or 2nd session for each of the four conditions (Uncrossed × Active, Uncrossed × ROS, Crossed × Active, Crossed × ROS). At a glance, the attractivity strength is remarkable in the Crossed × ROS condition, regardless of the order. A Three-Way within-participants ANOVA revealed a significant main effect of all three factors; 1st vs. 2nd: 0.187 vs. 0.262, *F*_(1, 35)_ = 5.93, *p* < 0.03; Uncrossed vs. Crossed: 0.135 vs. 0.314, *F*_(1, 35)_ = 13.69, *p* < 0.001; Active vs. ROS: 0.183 vs. 0.266, *F*_(1, 35)_ = 6.65, *p* < 0.02 but the interactions among the above three factors were not significant. The significant effect of the order implies that the participants learned to induce the illusion stronger with a passage of time even though the strong effect was only found in the Crossed × Active condition. The effect of resting the palm of the right hand on surface was siginificant but also limited to the Crossed condition during the 1st session. In contrast, the effect of crossing hands on attractivity was strongly general independently of the other factors, as is indicated in the low *p*-value.

### 3.2. Directivity

Figure 5B plots an average of the directivity for each of the four hand postures. A Three-Way ANOVA revealed the significant effect of two factors; 1st vs. 2nd: 0.169 vs. 0.075, *F*_(1, 35)_ = 4.92, *p* < 0.04; Uncrossed vs. Crossed: 0.217 vs. 0.027, *F*_(1, 35)_ = 6.02, *p* < 0.02; Active vs. ROS: 0.143 vs. 0.101, *F*_(1, 35)_ = 1.01, n.s., but the interactions among the above three factors were not significant. The detailed analysis compared 72 of the directivity values (collected from 36 participants × 2 sessions for each hand posture) with 72 zeroes using Welch's one-tailed *t*-test, revealing that the directivity significantly trended toward the positive sign when hands are uncrossed [Active: *t*_(71)_ = 5.21, *p* < 0.001; ROS: *t*_(71)_ = 4.45, *p* < 0.001], but demonstrating no significant trend when hands are crossed [Active: *t*_(71)_ = 1.25, n.s., ROS: *t*_(71)_ = 0.81, n.s.]. The results indicate that the PD of the administrating hand tends to be greater than that of the receptive hand (admin-dominant asymmetry) in the Uncrossed condition whereas that asymmetry almost completely vanishes in the Crossed condition. However, it does not mean that an asymmetrical PD does not occur in the Crossed condition at an individual level.

#### 3.2.1. Relationship between directivity and attractivity

**Figure 6:**
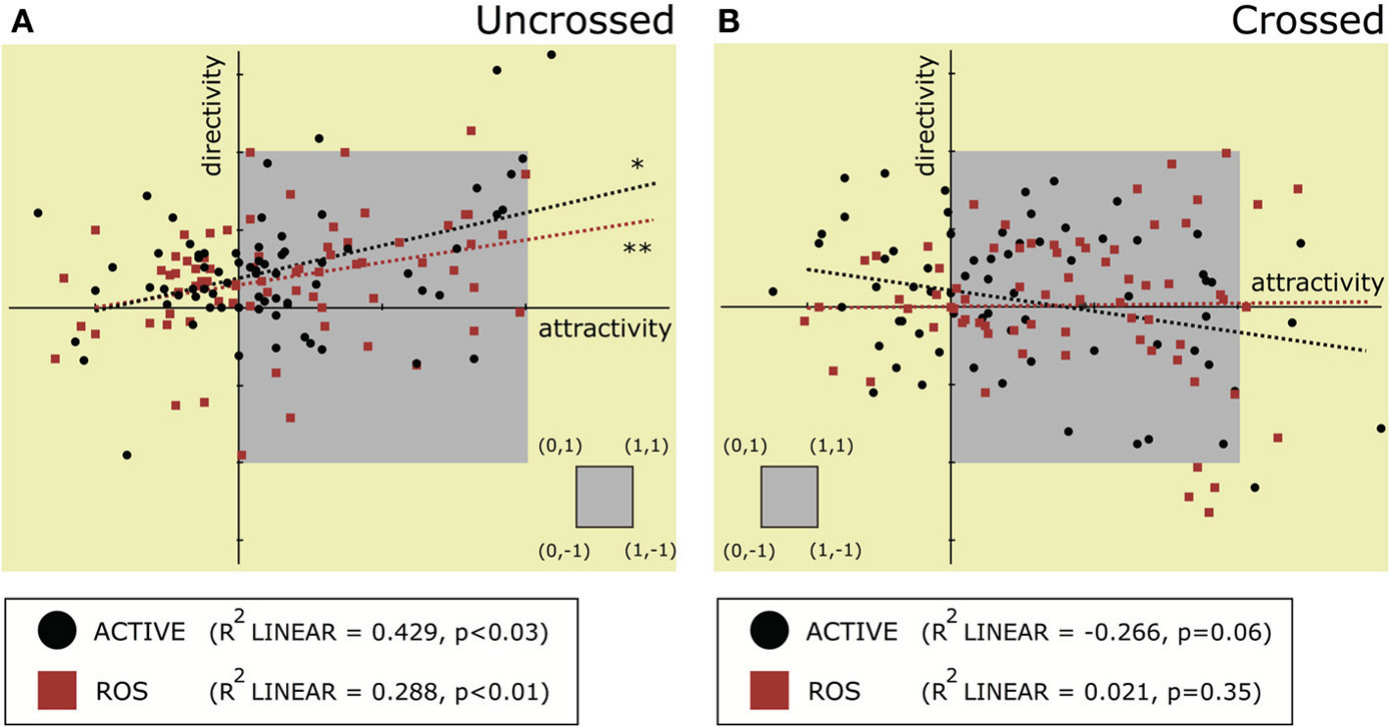
**Correlation diagram between attractivity and directivity in four types of hand postures**. Diagram **(A)** depicts the condition when hands are uncrossed, and **(B)** that when hands are crossed. In the diagrams, each small black dot represents data from the Active condition, and each red square represents data from the ROS condition. The gray rectangle indicates the area satisfying 0 < attractivity < 1 and −1 < directivity < 1. Asterisks indicate that there is a significant correlation between two variables (with Spearman's correlation test, ^*^p < 0.05, ^**^p < 0.01).

Figure 6 plots a correlation diagram between the attractivity and the directivity for each of the four hand postures. In Uncrossed × Active or Uncrossed × ROS, directivity had a moderate positive correlation with attractivity, meaning that a strengthened PD does not undermine the admin-dominant asymmetry (Spearman with *n* = 72, Uncrossed × Active: *r* = 0.238, *p* < 0.03; Uncrossed × ROS: *r* = 0.314, *p* < 0.01). In contrast, there is no specific trend of the correlation in Crossed × Active or Crossed × ROS in the entire group (Spearman with *n* = 72, Crossed × Active: *r* = −0.185, n.s.; Uncrossed × ROS: *r* = 0.046, n.s.). The significant effect of the order on the directivity may be mainly caused by such a moderately negative inclination of Crossed × Active (the attractivity in Crossed × Active jumped at the 2nd session, as can be seen in Figure 5A). Notably, each individual result demonstrated the existence of various types of the PDs in the region of strong attractivity, such as the admin-dominant (positive directivity) PD, the symmetric (close-to-zero directivity) PD, and the receptor-dominant (negative directivity) PD. Thus, the correlation absence in the Crossed condition solely resulted from the pattern of body images on the STI being diversified drastically.

### 3.3. Agreement ratings

Figure 5C compares the average of each questionnaire's agreement rating in each of the four conditions. At a glance, the illusion statement (Statement 1) received a stronger agreement rating than the other control statements. Analysis of a One-Way ANOVA of a factor on the statement pooled and averaged all agreement ratings of that statement for all eight sessions for every participant. The analysis revealed a significant effect on the statement [*F*_(4, 140)_ = 29.3, *p* < 0.001]. Multiple comparisons demonstrated that participant agreement rating for the illusion statement was significantly larger than that for all other statements (*p* < 0.001), and the agreement rating for statement 5 was significantly lower than that for statements 1 and 3–4 (*p* < 0.001). The figure reveals that the agreement rating for statements 1 and 4 was remarkably larger in Cross × ROS than those for the other hand postures. A Three-Way ANOVA revealed the significant effect of all the factors in statement 1 [1st vs. 2nd: *F*_(1, 35)_ = 7.58, *p* < 0.01; Uncrossed vs. Crossed: *F*_(1, 35)_ = 6.01, *p* < 0.02; Active vs. ROS, *F*_(1, 35)_ = 6.05, *p* < 0.02] and the significant effect of one factor in statement 4 [Uncrossed vs. Crossed, *F*_(1, 35)_ = 5.49, *p* < 0.03], while interactions among three factors were not significant in each statement.

#### 3.3.1. Relationship between attractivity and agreement ratings

**Figure 7:**
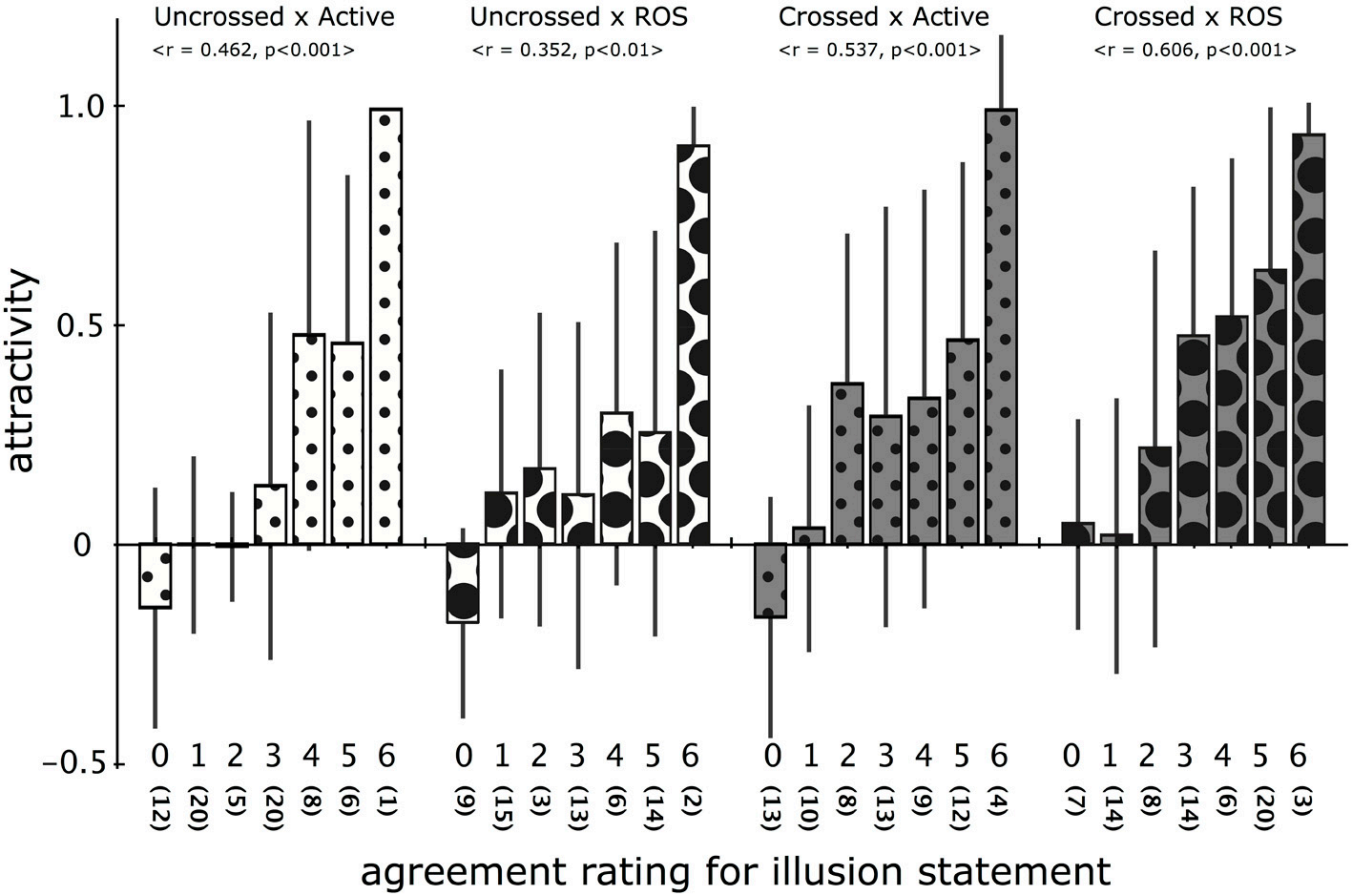
**Relationship between agreement rating for illusion statement and average attractivity in four types of hand postures**. Each average of attractivity was calculated from all sessions involving the same agreement rating (0–6) for the illusion statement. The number in parentheses indicates how many sessions produced the corresponding agreement rating, and the statistics depict the result of the Spearman correlation test. Error bars indicate standard error.

Figure 7 compares the average of attractivity values pooled from all sessions involving the identical agreement rating (0–6) for the illusion statement. As the graph obviously indicates, the attractivity strongly and positively correlated with subjective illusion strength (Spearman with *n* = 72, Uncrossed × Active: *r* = 0.467, *p* < 0.001; Uncrossed × ROS: *r* = 0.352, *p* < 0.01; Crossed × Active: *r* = 0.537, *p* < 0.001; Crossed × ROS: *r* = 0.606, *p* < 0.001). Specifically, the attractivity was near or less than zero when the rating was 0 or 1, whereas it jumped to near 0.5 for the ratings of 4 or 5. In sessions with the rating of 6 (“very strongly agree”), which had few samples, the attractivity was near 1.0 without exception, meaning that both index fingers touched each other on the level of the body image.

## 4. Results—experiment II

Prior to analysis, the attractivity and directivity of each participant were calculated for each of the three conditions using the common (*R*_*x*_, *A*_*x*_) coordinates where five sets of camera positions for the felt location of both index fingers at the pre-illusion stage were pooled and averaged per body midline alignment to restrain dispersion.

### 4.1. Attractivity

**Figure 8:**
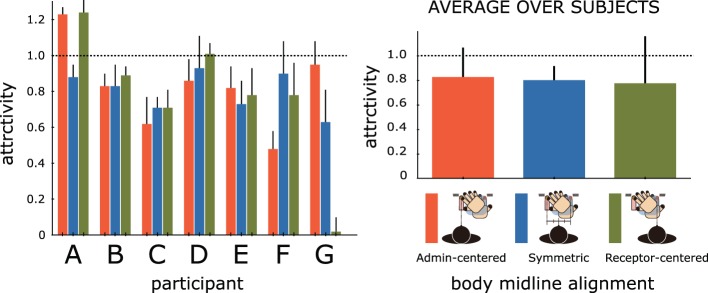
**Results of the average attractivity of seven “illusion-sensitive” participants for three types of body midline alignments in Experiment II**. Three colors depict where the body midline is aligned in the session; it is aligned with the administrating (right) hand in the Admin-centered condition, with the receptive (left) hand in the Receptor-centered condition, and between both hands in the Symmetric condition where hands are crossed. The left graph shows the average attractivity of each participant and the right graph shows the average over all participants, where error bars indicate standard error.

Figure 8 depicts the average attractivity for each of the three body midline alignments for the seven “illusion-sensitive” participants, revealing that almost all participants experienced strong attractions between two fingers independently of the body midline alignment. The only exception was participant G in the Receptor-centered condition where the attractivity mean was 0.02, suggesting no experience of the STI. The average attractivity for the seven participants was 0.80, the highest average was 1.12 (participant A), and the lowest was 0.53 (participant G, but it jumps to 0.79 when excluding five sessions in the Receptor-centered condition). The average attractivity for the Crossed × ROS condition in Experiment I was 0.88 among the seven “illusion-sensitive” participants but 0.26 among the other 29 participants. The level of attractivity obtained in the group of the students who strongly felt the illusion in Experiment I remained much higher in Experiment II than that of the other participants, indicating that a sensitivity for the STI is quite participant specific. The graph also shows the average attractivity over all participants for each body midline, demonstrating that the effect of body midline alignment on attractivity strength is limited highly. Apparently, we could find no specific alignment to robustly produce a stronger attraction than the other conditions among the seven participants, which was supported by a One-Way ANOVA showing that the body midline alignment did not have a significant effect on the level of the attractivity [*F*_(2, 12)_ = 0.12, n.s.].

### 4.2. Directivity

**Figure 9:**
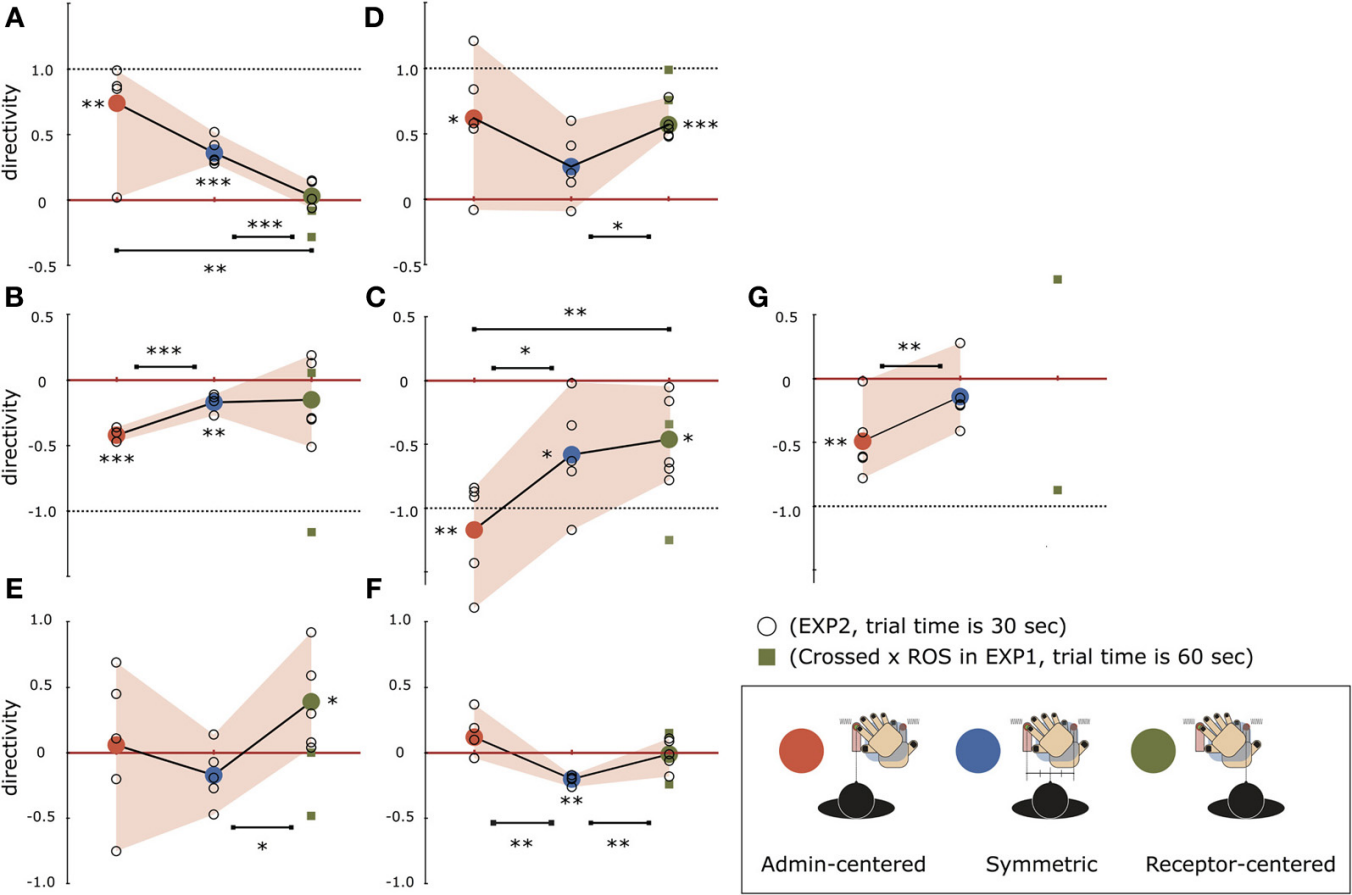
**Results of the directivity distribution of seven (A–G) “illusion-sensitive” participants for three types of body midline alignments in Experiment II**. Each body midline alignment experiment contains five sessions. The individual data from each session are plotted as a small blank circle, while a large colored circle represents the average attractivity for each body midline alignment. The directivity values in the Crossed × ROS condition of Experiment I (including two sessions every participant) are depicted together with those in Experiment II by a small square, in the region of the Receptor-centered condition. Data of participant G in the Receptor-centered condition of the Experiment II was excluded because of low attractivity. Asterisks indicate that there is significant difference of directivity between two body midline alignments or that a series of attractivity in each body midline alignment are significantly different from zero (with one-tailed Welch's *t*-test, ^*^p < 0.05, ^**^p < 0.01, ^***^p < 0.001).

Figure 9 maps the distribution of the directivity values for the three body midline alignments of each of the seven participants (data of participant G in the Receptor-centered condition was excluded because of low attractivity). Participants A and D exhibited an admin-dominant PD revealed by significant positive directivity [one-tailed Welch's *t*-test comparison with zeroes, A: *t*_(14)_ = 4.24, *p* < 0.001; D: *t*_(14)_ = 5.82, *p* < 0.001]. Participant A exhibited relatively symmetric PD in the Receptor-centered condition whereas admin-dominant asymmetry strengthens when the body shifts leftward. Specifically, the directivity was more than 0.8 in four out of five sessions in the Admin-centered condition. Participant D maintained the admin-dominant PD independently of the body midline alignments, but a degree of the asymmetry depended on the alignments. In contrast, participants B, C, and G completely exhibited receptor-dominant PD revealed by significant negative directivity [B: *t*_(14)_ = 4.93, *p* < 0.001; C: *t*_(14)_ = 6.26, *p* < 0.001; G: *t*_(9)_ = 3.57, *p* < 0.01]. In participant B, the tendency of having the receptor-dominant PD was fairly robust in both Symmetric and Admin-centered conditions though the intensity was not particularly large. Participant C exhibited significant receptor-dominant PDs in all three body midline alignments. Especially in the Admin-centered condition, the felt location of the receptive (left) hand shifted toward the place where the administrating (right) hand was placed, whereas the felt location of the administrating hand shifted even farther leftward from the original position, resulting in the high level of the negative directivity. Negative directivity was observed for almost all sessions in participant G but was especially robust in the Admin-centered condition. It is interesting that the receptor-dominant asymmetry in these three participants was maximized when the body midline was aligned at the administrating hand (Admin-centered condition). Finally, participants E and F did not exhibit specific asymmetry in the PD overall, unlike the aforementioned five participants. The directivity observed in participant E strongly varied among all body midline alignments. Specifically, it varied in the positive region for the Receptor-centered condition but mainly in the negative region for the Symmetric condition. Such randomness increased in the Admin-centered condition where both clearly positive and negative directivity (0.69 and −0.75) were observed during the five sessions. Conversely, participant F did not exhibit such a scattered distribution of the directivity. Almost all the directivity was relatively close to zero value, even though there was a significant receptor-dominant PD in the Symmetric condition.

## 5. Discussion

### 5.1. Sense of self-touch and proprioceptive drift

The agreement rating for the illusion statement and the total distance of the PD has been generally used to estimate the strength of the RHI and STI. It has been demonstrated that the PD distance positively correlates with the agreement rating (e.g., Ehrsson et al., 2005; Longo et al., 2008; Lopez et al., 2010; Kalckert and Ehrsson, 2012). However, it has been also known that it is uncertain whether the PD is a component of the illusion (Rohde et al., 2011) because the PD in the RHI can occur even with asynchronous contact (Holmes et al., 2006) or merely by looking at the prosthetic hand exposed to a beam of light (Durgin et al., 2007). Our experimental results clearly demonstrate that the STI involves considerable PD. Figure 7 demonstrates that the attractivity observed during sessions with the agreement rating 0–1 for the illusion statement is near or less than 0 and that considerable attraction should involve moderate experience of the illusion (agreement rating: 3). There were 110 sessions with the illusion rating of 0–1, whereas only four instances produced attractivity of more than 0.5, and no cases produced attractivity of more than 0.7 (We call such a situation “full attraction.”). These results demonstrate that it is quite valid to regard the PD beyond a certain level as proof of the subjective STI experience.

However, it is also important to confirm that a backward presumption is not valid as follows. Among 142 sessions with the illusion rating of 3–5, 47 instances produced the attractivity less than 0.1 (33%). Many participants in this group commented that they actually felt the sense of the self-touch but obviously felt that the two fingers remained separated, indicating that the sense of the STI can occur even without canceling a spatial inconsistency. In this sense, the PD is apparently not required for the STI. In contrast, full attraction robustly occurred in the sessions with the strongest illusion rating (6) even with few samples (10 instances in five participants). The aforementioned consideration suggests the assumption that the STI has two stages in the quality of the illusion. Specifically, the PD is not required for the STI in a broad sense whereas an intense STI in a narrow sense should involve the full attraction in the PD.

### 5.2. Anatomic plausibility and anatomic dissonance

Experiment I revealed that resting the palm of the administrating hand on the surface (ROS operation) has a significantly positive effect in inducing the STI. It is appropriate to individually discuss the effect of the ROS operation for the Uncrossed and Crossed conditions.

First, we focus on the Uncrossed condition where an increased rate of the illusion rating by the ROS operation was roughly 27%: 2.19 (Uncrossed × Active) vs. 2.58 (Uncrossed × ROS), *t*_(35)_ = 2.28, *p* < 0.03, and that of the attractivity was roughly 30%; 0.118 vs. 0.152, *t*_(35)_ = 1.20, n.s. (with a two-tailed paired *t*-test where each measure was pooled and averaged per hand posture). Grounding a body part on a location should increase the reliability of the proprioceptive sense, resulting in an expectation that the variability of the body image would decrease the illusion based on the proprioception reliability paradigm. However, deduction does not accord with this experiment's result. Concerning this point, it is informative that a few participants commented that they felt as if the right index finger extended slightly toward the left index finger. The direction in which the right index finger (contacting the rubber finger) pointed was rather downward (the direction toward the bottom of the desk) in the Active condition but leftward on the desk (the direction toward the back of the left index finger) in the ROS condition. Thus, not only shifting the entire right hand toward the left hand (PD) but also extending the right index finger toward the left hand (anatomic extension) can be used as a means of creating the illusion of self-touch in the Uncrossed × ROS condition. Considering that the contribution of the administrating hand to the PD rather decreased from the ROS operation [the directivity is 0.244 (Uncrossed × Active) vs. 0.190 (Uncrossed × ROS), *t*_(35)_ = 1.61, n.s.], the positive effect of the ROS operation on the subjective illusion strength was not caused by the change of the proprioceptive variability of the administrating hand but rather by the positive contribution of anatomic plausibility based on the similarity between the actual hand posture and the potential body image on the STI (as discussed in White and Aimola Davies, 2011).

The following discussion describes how the ROS operation contributes to inducing the STI in the Crossed condition. The ROS operation increased the illusion rating by 12%: 2.64 (Crossed × Active) vs. 2.97 (Crossed × ROS), *t*_(35)_ = 2.06, *p* < 0.05, which was smaller than that in the Uncrossed condition; however, the increase rate of the attractivity reached as much as 53%: 0.248 vs. 0.380, *t*_(35)_ = 3.26, *p* < 0.01. Thus, the mechanism producing the positive effect of the ROS operation in the Crossed condition seems different from that in the Uncrossed condition. Specifically, extending to the right index finger toward the direction in which it points causes a separation from the position of the left index finger. The degree of the separation is especially large in the Crossed × ROS condition because the right index finger is entirely directed leftward. This means that the ROS operation is disadvantageous in inducing the STI in not only proprioceptive variability but also anatomic plausibility when the hands are crossed.

What factors can reverse the negative effects in the Crossed × ROS condition? To address this issue, the authors propose a new concept called “anatomic dissonance” as follows. In the Crossed × ROS condition, the palms of both hands were ROS where the right hand was placed on the acrylic stand 3 cm above the left hand so that both hands were not touching. Thus, the stand functioned as a partition by vertically dividing the hands by a short distance, which made quite an unusual spatial situation for both hands. It is important to confirm that both hands should touch each other if there is no artificially inserted partition. It is probable that closing the eyes and hearing white noise makes a participant's attention to the partition unstable, which would enhance the feeling of contradiction between the proprioceptive sense and the sense of touch: “Two hands are sufficiently close to touch each other, but I cannot feel such a sense of touch.” We call such a situation anatomic dissonance after a well-known concept “cognitive dissonance.” In the same way that cognitive dissonance forces the alteration of one fact in mutually contradictory information (Festinger and Carlsmith, 1959), anatomic dissonance can compel either the proprioceptive sense or the touch sense to change to cancel the contradiction. The proprioceptive sense is obviously more flexible than the sense of touch; it would be difficult to invent a new touch sensation on the back of the left hand. Therefore, changing the body image toward the uncrossed posture (consistent with the self-touch situation) is a sensible resolution.

Such an effect may occur to some extent even in the Crossed × Active condition. When the palm of the right hand is active on the surface (Active), crossing hands increased the illusion agreement rating by 21%: 2.19 (Uncrossed × Active) vs. 2.64 (Crossed × Active), *t*_(35)_ = 2.19, *p* < 0.04, and the attractivity by more than double: 0.118 vs. 0.248, *t*_(35)_ = 2.47, *p* < 0.02. This comparison is sufficient to speculate that there was an essential change in the process of inducing the STI between the Uncrossed and the Crossed conditions though the illusion level in the Crossed × Active condition was lower than that in the Crossed × ROS condition. In the Crossed × Active condition, the four fingers in the right hand were wrapped, and the right elbow was held in the air so that the right hand would not touch the back of the left hand. The wrapped fingers were nonetheless close to the back of the left hand because the distance between two index fingers was as little as 10 cm. It generally seems quite rare in the hands-crossed condition that the hands are not touching when they are so close to each other. Therefore, the Crossed × Active condition may also induce the effect caused by anatomic dissonance.

### 5.3. Is the proprioceptive reliability paradigm effective?

In the previous section, the authors regarded the anatomic dissonance as a main cause of the PD increasing in the Crossed condition. The following discussion examines how the proprioceptive variability change by crossing hands affects the PD, which could be another cause. According to the recent research of Rincon-Gonzalez et al. (2011), a hand's proprioceptive uncertainty increases when the hand is across the body midline (note that the other hand rested on a chair's armrests; both hands were not necessarily crossed). Because crossing hands necessarily makes one hand go across the body midline, it is worth examining the possibility that a decrease of the hand's proprioceptive reliability affects the PD increase. It is, however, difficult to assert that such an effect played an important role in increasing the PD. In Experiment I with the body midline aligned along the left (receptive) hand, only the right (administrating) hand was crossing the body midline in the Crossed condition (Crossed × Active or Crossed × ROS). Accordingly, the PD's increase should be explained as an effect of the proprioceptive reliability decrease of the right (administrating) hand if the reliability decrease is regarded as a main cause of the PD increase. This consideration causes an expectation that the admin-dominant asymmetry generally observed in the Uncrossed condition would be strengthened in the Crossed condition. The result was not, however, consistent with this expectation; the admin-dominant asymmetry was instead canceled by the appearance of the receptor-dominant PD in the Crossed condition. In addition, the result of Experiment II throws an essential doubt upon proprioceptive reliability's effect on the PD. In Experiment II, the Receptor-centered condition should produce stronger directivity than the Admin-centered condition on the basis of the proprioceptive reliability paradigm because the hand across the body midline is the receptive (left) hand in the Admin-centered condition and an administrating (right) hand in the Receptor-centered condition. Only one (participant C) among the seven participants significantly exhibited such a tendency, whereas participant A significantly exhibited the opposite tendency. Thus, it is valid to assume that the decrease of the proprioceptive reliability due to the contralateral effect is not a main factor to induce a stronger PD in the Crossed condition.

The proprioceptive reliabilities of both hands might drastically decrease not simply by a single hand crossing the body midline but by a mutually complex effect involved with crossed hands, which would uphold the validity of the proprioceptive reliability paradigm. It is well known that crossing hands (Yamamoto and Kitazawa, 2001; Heed et al., 2012) or unnatural hand configuration (Haggard et al., 2006; Hong et al., 2012) itself reduces basic spatial discrimination ability (Heed and Azañón, 2014 for a review). In addition, this ability might deeply depend on body-image modulation (Azañón and Soto-Faraco, 2007). It is interesting to assume that such a mechanism reduces the proprioceptive reliability of the both hands. To demonstrate this effect, further research on the proprioceptive sense with hands crossed is required.

### 5.4. What caused asymmetrical proprioceptive drift?

The admin-dominant asymmetry on the PD in the uncrossed condition has been robustly observed in a series of studies by White et al. (2011) and Aimola Davies et al. (2013). In their experiments, the administrating (right) hand was aligned along the body midline, and the touch to a rubber hand was administered via a writing brush. In the uncrossed condition of our experiments, the receptive (left) hand was aligned with the body midline, and the touch to the rubber finger was directly administered by a right index finger. Regardless of this difference, our experiment accurately reproduced the trend of PD directivity seen in the past work. Thus, the uncrossed hand condition involves a fairly strong trend where the administrating hand is dominant over the receptive hand in the PD. Moving a body image corresponding to a body part that is found to be physically stationary seems difficult, considering that phantom pain is essentially caused by a “learned paralysis” (Ramachandran and Hirstein, 1998). Similarly, it is natural to assume that body-image variability becomes feeble when the corresponding body part is completely stuck to the surface (ROS) but strengthened when it is in contact motion. Specifically, the right forearm and administrating right hand are prone to be directed toward the receptive left hand in the uncrossed condition. It is probable that a touching movement functions as a good catalyst for sliding the administrating right hand toward the receptive left hand on the level of the body image in such an anatomically plausible condition.

In contrast, the distribution of the PD's directivity in the Crossed condition exhibited considerable disorder. First, Experiment I revealed that there was no specific trend on the directivity in the Crossed condition among the 36 participants. Each exhibited a different type of PD power balance, typically categorized into three types: admin-dominant PD, symmetric PD where both hands contribute relatively equally to the PD in terms of the size of the intensity in the directivity, and receptor-dominant PD. Such diversity was symbolically reproduced in Experiment II involving seven “illusion-sensitive” participants who can robustly experience the illusion with a strong attraction. Two participants exhibited the admin-dominance trend, and three participants the receptor-dominance trend. Among their 15 sessions, instances where the sign of the directivity was different from the participant-specific dominant sign were quite rare (+/−; A:14/1, B:2/13, C:0/15, D:13/2, G:1/9). Nonetheless, the body midline alignment strongly determined the intensity of participant-specific directivity; the aforementioned five participants (as well as the remaining two) actually exhibited a significant change of directivity among at least two body midline alignments. Next, we focus on two participants (E and F) who did not exhibit a specific trend of directivity during the entire 15 sessions. The intensity of directivity in the sessions with participant F was small regardless of whether the sign was positive or negative even though the trend toward the negative directivity was significant in the Symmetric condition. Thus, participant F exhibited a unique trend with non-directional PD (what we call symmetric PD) throughout the three body midline alignments. What we can learn from the directivity distributions of these six participants is that each participant likely exhibits the participant-specific trend of PD directivity when hands are crossed, and it remains relatively stable over multiple body midline locations and time.

However, this theory is not valid for participant E, who exhibited a fairly complex PD trend over 15 sessions. On balance, the trend was receptor-dominant when the body midline was aligned between both hands (in sessions 6–10) but became admin-dominant when it was aligned with the receptive hand (in sessions 11–15). Even more interesting, the directivity was −0.75 (attractivitiy = 0.66) in the second session but 0.69 (attractivity = 1.00) in the fourth session in the Admin-centered condition (in sessions 1–5), meaning that only a short time course can trigger the essential change of the body image. Thus, participant E obviously did not have a specific PD directivity trend, unlike the other participants. Such an instability in a single person was not exclusive to participant E. As one example, participant G exhibited two different body images for the Crossed × ROS condition in Experiment I such that the directivity was 0.76 (attractivity = 0.65) in the second session and −0.84 (attractivity = 1.16) in the fifth session, as shown in Figure 9. These observations suggest that crossing hands would involve a chaotic mechanism in the process of producing the body image related to the STI. That is, the six participants exhibiting the specific trend of directivity in Experiment II would destabilize the directivity when placed in a different context (Actually, participants B and E showed the strong negative directivity in Experiment I which was not observed during five sessions of the Receptor-centered condition in Experiment II, as shown in Figure 9).

It would be useful to bring the concept of “anatomic plausibility” again into the discussion to examine why the admin-dominant asymmetry strongly observed in the Uncrossed condition disappeared in the Crossed condition. As previously mentioned, the anatomic plausibility is drastically reduced when hands are crossed because the direction of the right forearm and index finger point is away from the left hand. It is difficult to speculate that such a spatially discordant action by the administrating right hand works well in drifting the body image of the administrating hand toward the receptive hand (rightward). Concerning this point, research has suggested that the speed of mental rotation toward a picture of one hand is maximized when the direction of rotating a handle-type joystick (simultaneously conducted) is in accord with the turning angle of the hand in the picture (Wexler et al., 1998; Wohlschläger, 1998). In this context, the administrating hand's pointing rightly toward the receptive hand is probably quite useful to drift the administrating hand toward the receptive hand. If the disappearance of the admin-dominant asymmetrical PD in the Crossed condition was thus caused by undermining the anatomical plausibility, conversely, it is valid to hypothesize that the admin-dominant asymmetry in the uncrossed condition was essentially caused by the effect of anatomical plausibility, not by the difference between both hands' proprioceptive reliabilities.

## 6. Conclusion

This study primarily examined the effect of two factors in inducing the STI: (1) whether hands are uncrossed or crossed and (2) whether the administrating (right) hand is resting or active on the surface. Results demonstrated that both crossing and resting operations have significantly positive effects on the STI. What is especially notable is that crossing hands increased more than doubles the total distance of the PD and diversified its power balance. Our discussion indicates that a difference of the anatomic structure rather than a difference of the proprioceptive reliability strongly affects the production of this effect. In the analysis, two concepts were introduced to interpret the experimental results concerning the anatomical structure: “anatomic plausibility” and “anatomic dissonance.” Anatomic plausibility produces a positive effect when the administrating hand faces toward the receptive hand, causing the strong admin-dominant asymmetry in the uncrossed condition. In contrast, “anatomic dissonance” induces a strong PD (regardless of whether it is admin-dominant or receptor-dominant) in the crossed condition where anatomic plausibility is undermined completely. This effect is strong especially when the palm of the right hand is resting on the stand slightly above the left hand because an absent sense of touch (on the back of the left hand) contradicts information obtained from the proprioceptive sense.

This study has addressed only one aspect of the disappearance of anatomic plausibility to explain why the pattern of the PD was diversified in the crossed condition. Although it cannot explain at all why the power balance of the PD is relatively unique to each participant or why a participant can exceptionally produce a different type of the PD in the identical posture, this explanation can accurately describe why the admin-dominant trend disappears. The dominance of the PD may also be influenced by attentional modulation as the study of binocular rivalry suggests that attention partially modulates the process of determining the perceptual dominance (Dieter and Tadin, 2011; Paffen and Alais, 2011). The important issue to examine the cognitive process involved in producing the body image of the STI in a chaotic manner when hands are crossed remains for future research.

### Conflict of interest statement

The authors declare that the research was conducted in the absence of any commercial or financial relationships that could be construed as a potential conflict of interest.
